# Models of Eucalypt phenology predict bat population flux

**DOI:** 10.1002/ece3.2382

**Published:** 2016-09-21

**Authors:** John R. Giles, Raina K. Plowright, Peggy Eby, Alison J. Peel, Hamish McCallum

**Affiliations:** ^1^ Environmental Futures Research Institute Griffith University Brisbane Queensland 4111 Australia; ^2^ Department of Microbiology and Immunology Montana State University Bozeman Montana 59717; ^3^ School of Biological, Earth, and Environmental Sciences University of New South Wales Sydney New South Wales 2052 Australia

**Keywords:** Foraging ecology, fruit bat, Hendra virus, henipavirus, machine learning, population dynamics, *Pteropus*, spillover, viral prevalence

## Abstract

Fruit bats (Pteropodidae) have received increased attention after the recent emergence of notable viral pathogens of bat origin. Their vagility hinders data collection on abundance and distribution, which constrains modeling efforts and our understanding of bat ecology, viral dynamics, and spillover. We addressed this knowledge gap with models and data on the occurrence and abundance of nectarivorous fruit bat populations at 3 day roosts in southeast Queensland. We used environmental drivers of nectar production as predictors and explored relationships between bat abundance and virus spillover. Specifically, we developed several novel modeling tools motivated by complexities of fruit bat foraging ecology, including: (1) a dataset of spatial variables comprising Eucalypt‐focused vegetation indices, cumulative precipitation, and temperature anomaly; (2) an algorithm that associated bat population response with spatial covariates in a spatially and temporally relevant way given our current understanding of bat foraging behavior; and (3) a thorough statistical learning approach to finding optimal covariate combinations. We identified covariates that classify fruit bat occupancy at each of our three study roosts with 86–93% accuracy. Negative binomial models explained 43–53% of the variation in observed abundance across roosts. Our models suggest that spatiotemporal heterogeneity in Eucalypt‐based food resources could drive at least 50% of bat population behavior at the landscape scale. We found that 13 spillover events were observed within the foraging range of our study roosts, and they occurred during times when models predicted low population abundance. Our results suggest that, in southeast Queensland, spillover may not be driven by large aggregations of fruit bats attracted by nectar‐based resources, but rather by behavior of smaller resident subpopulations. Our models and data integrated remote sensing and statistical learning to make inferences on bat ecology and disease dynamics. This work provides a foundation for further studies on landscape‐scale population movement and spatiotemporal disease dynamics.

## Introduction

In recent years, there has been an increase in the emergence of bat‐borne viral pathogens in humans and livestock (Calisher et al. 2006; Halpin et al. 2007; Drexler et al. 2012; Luis et al. 2013; Han et al. 2015). Many of these are negative‐stranded RNA viruses, which originate in the nectarivorous and frugivorous mega‐bats of the tropics and subtropics (Filoviruses, Coronaviruses, Lyssaviruses, Henipaviruses (Chua et al. 2000; Leroy et al. 2005; Peel et al. 2012; Banyard et al. 2013). Despite the different host–pathogen relationships in this group of viruses, they exhibit similar patterns of annual or interannual patterns of infection and spillover. Viral infections have been present in bats over evolutionary timescales, and bats have been historically sympatric with humans throughout Africa, Asia, and Australia. But the preponderance of emergence events have occurred within the last 20 years (Woolhouse et al. 2005; Woolhouse and Gaunt 2007). Outbreaks of Hendra virus in 1994, Nipah virus in 1998, Ebola virus in 2014 have been associated with significant landscape changes in the years prior (Bradshaw 2012; Pulliam et al. 2012; Bausch and Schwarz 2014), but limited data exist on how bat populations are influenced by such changes, making it difficult to ascertain the role of landscape change in bat‐borne disease emergence.

We use the tropical and subtropical fruit bat species of eastern Australia as an example of how a mechanistic link can be made between food resource phenology and bat population response in the context of Hendra virus disease ecology. Endemic to Australia, Hendra virus resides in its reservoir host, *Pteropus* fruit bats and periodically infects horses, and subsequently, humans (Halpin et al. 2000, 2011; Field et al. 2011). The emergence of Hendra virus is a good study system for questions about host population dynamics and disease emergence because: (1) Reliable data have been collected on viral prevalence, spillover events (transmission from reservoir host to secondary host such as livestock and humans), and host population distributions; and (2) Hendra virus‐fruit bat ecological dynamics can provide insights into other important bat‐borne viral zoonoses which have emerged in Africa and Asia. Since its emergence in 1994, Hendra virus spillover occurs sporadically and aseasonally in the tropics, but interannually during winter in the subtropics (Plowright et al. 2015). This curious pattern of Hendra virus spillover has been explored through spatiotemporal surveys of viral prevalence and seroprevalence in bats (Plowright et al. 2008; Breed et al. 2011; Field et al. 2011, 2015b) and modeling studies of virus survival under different environmental conditions (Martin et al. 2015). Yet, the less explored host population dynamics can have a profound impact on the ecology underlying Hendra virus prevalence and spillover. Hypotheses proposed to explain pulses of infection in bat populations suggest that viral dynamics are intrinsically linked to fluctuations in bat populations (flux) or the food resources that drive these fluctuations, whether through population density effects on transmission, or environmental stress effects on viral excretion (Plowright et al. 2015). The precise manner in which fruit bat population density influences viral dynamics is uncertain, and models of bat population flux are necessary to understand observed patterns in viral prevalence or spillover.

The unique foraging behavior of fruit bat populations presents some challenges to modeling population dynamics. There are four species of Australian fruit bats: the little red flying fox (*Pteropus scapulatus*), the spectacled flying fox (*P. conspiculatus*), the black flying fox (*P. alecto*), and the gray‐headed flying fox (*P. poliocephalus*) (see Fig. S1 for distributions (Ratcliffe 1931). Hendra virus and anti‐Hendra virus antibodies have been detected in all four species; however, *P. alecto* is suspected to be the most important source of spillover (Daniels et al. 2007; Smith et al. 2014; Edson et al. 2015; Field et al. 2015b; Goldspink et al. 2015). Spatial distribution and behavior can be highly variable across species and individuals, ranging from sedentary to entirely migratory behavior (Eby 1991; Smith et al. 2011; Roberts et al. 2012b). Individuals typically forage within 20–30 km of their day roost, but have been observed traveling over 100 km in a night or many hundreds of kilometers within a few days (Roberts et al. 2012b). At the population level, movement among roosts can be continuous at low levels or involve the relocation of entire populations to a new roosting site depending on foraging conditions or disturbance (Eby et al. 1999; Shilton et al. 2008; Roberts et al. 2012b).

Fruit bat populations rapidly disperse and coalesce, redistributing large numbers of individuals across the landscape to forage on pollen, nectar, and capsular fruit of Myrtaceous trees, especially *Eucalyptus* and *Corymbia* (generally referred to as Eucalypts), *Melaleuca*, and *Banksia* (Courts 1998; Eby 1998, 2008; Birt 2004; Eby and Law 2008). Flexible population structure is manifested by low spatial cohesiveness and loosely defined social relationships, which can produce fission‐fusion type behavior (Aureli et al. 2008). Synchronized group movement occurs when environmental pressure, such as local resource depletion, migrating to unusually large pulses of nectar, or disturbances such as human aided dispersal and cyclones, reaches a threshold and precipitates a unanimous decision within and among groups (Eby et al. 1999; Conradt and Roper 2005; Shilton et al. 2008). As the majority of the fruit bat diet is comprised of pollen and nectar (Parry‐Jones and Augee 1991; Courts 1998), cycles of flowering and nectar production in Eucalypts drive a significant portion of bat population dynamics. Therefore, detecting phenological cycles in Eucalypts is necessary for building models of bat population flux.

A primary challenge is identifying variables that detect phenological cycles of flowering and nectar production across a landscape used by a population of bats. The Eucalypts of Australasia are broad‐leaf evergreens that undergo continuous cycles of growth and reproduction adapted for animal‐mediated pollination. Regional Eucalypt communities are often diverse, with unique responses to climate, which produce variable annual and interannual phenological cycles depending on the climate regime and species assemblage of the region (Law et al. 2000; Wilson 2003; Keatley and Hudson 2007). Remotely sensed reflectance and climate data that characterizes temporal changes in the local environment may enable detection of flowering and nectar production. However, to our knowledge, no studies have been published that directly link remotely sensed or climate data to nectar production. Fortunately, ample evidence in the literature provides sound basis for construction of proxy variables for flowering (Lindenmayer et al. 2015). For example, Eucalypt canopies experience a peak in leaf area during growth phases, which are then followed by decreased vegetative growth and initiation of the flowering process (Ashton 1975; Pook 1984; Pook et al. 1997). Changes in forest canopy structure and leaf physiology between these phenological cycles are discernible in remotely sensed spectral reflectance data and indices thereof (Coops et al. 1997; Datt 1998, 1999; Leuning et al. 2005; Ahl et al. 2006; Youngentob et al. 2012). Additionally, modeling studies show climatic factors, especially changes in temperature and precipitation in the months prior to flowering, are key predictors of the annual timing of flowering events (Keatley et al. 2002; Hudson et al. 2011a,b; Rawal et al. 2014a, 2015). Therefore, remotely sensed reflectance and climate data together serve as pertinent proxy variables for flowering and nectar production of Eucalypt forests.

Here, we develop a novel approach to predict bat population changes over time in response to food resource phenology. Previous research has analyzed trends in roost locations or zoonotic transmission events, within a presence‐only modeling framework, to identify the spatial distribution of bats and/or risk of disease transmission to humans (Peterson et al. 2004; Parsons et al. 2010; Hahn et al. 2014a,b; Pigott et al. 2014; Smith et al. 2014; Thanapongtharm et al. 2015). However, models of roost population abundance and occupancy remain unexplored, even though trends in abundance are important for understanding disease dynamics within bats and risk of transmission to humans. Models of roost occupancy are challenging because nectar pulses, which attract migratory bats, are driven by climatic conditions over various time lags (Keatley et al. 2002; Rawal et al. 2014a), and Eucalypt species assemblages are regionally diverse, so the timing and magnitude of nectar pulses is regionally unique (Keatley and Hudson 2007; Birtchnell and Gibson 2008). We address these challenges using proxies of nectar production that detect temporal patterns of general physiological states of Eucalypts (e.g., growth phases, bud formation, and flowering) to predict bat population flux. We develop a novel spatial sampling algorithm that relates bat population response to custom‐built proxies of nectar production and employ a statistical learning approach to identify optimal combinations of predictors. This study is part of a broader effort to characterize spatiotemporal bat population flux at the landscape scale and understand its relationship with viral prevalence and spillover.

## Materials and Methods

### Bat population data

**Figure 1:**
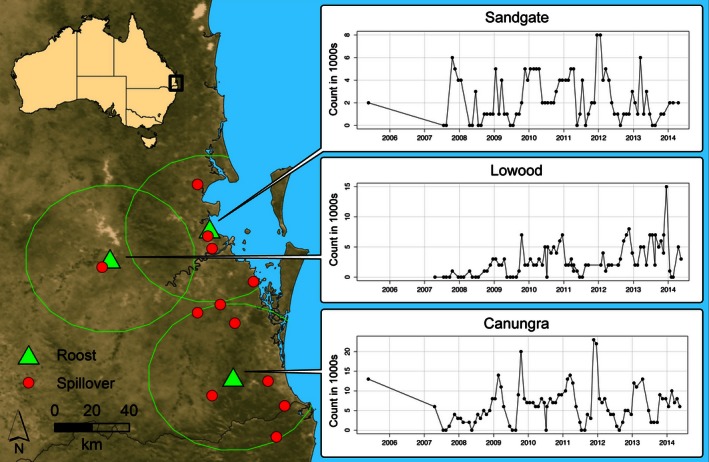
A map of our study area in southeast Queensland showing locations for three analyzed roosts (green triangles), maximum foraging radius (40 km), and locations of spillover events (red circles) that have occurred within the foraging radius of each roost. Time series of census counts display the recorded population counts at each location.

Data on bat population distribution come from the southeast Queensland flying fox monitoring program database, which was conducted by the Queensland Department of Environment and Heritage Protection. Estimated numbers of each species are recorded at “fly‐out” when bats are leaving the roost at dusk to forage. Census observations were recorded at approximately bimonthly intervals (with occasional irregularity) from 2005 to 2014 and display high variability, which appears qualitatively seasonal (Fig. 1). Three roosts were selected from the database located at Sandgate, Lowood, and Canungra (Fig. 1). These roosts were selected because they satisfied several criteria: (1) limited overlap within a 40 km foraging radius; (2) minimal interference from the migrations of the little red flying fox (*P. scapulatus*), which appear sporadically across SEQ in large nomadic groups of over 100,000; (3) sample sizes of census counts were large enough for rigorous cross‐validation; and (4) Hendra virus spillover events have been observed within their foraging radii. Based on similar foraging ecology, census counts of *P. alecto* and *P. poliocephalus* were combined to give the total bat population response used in our models.

### Spatial variables

Both spectral reflectance data and climate parameters were acquired and processed into variables designed specifically to detect both flowering events and nectar availability in Eucalypts. The sequence of Eucalypt leaf production, bud initiation, and anthesis is similar across species and produce changes in canopy structure, which can be detected via satellite imagery (Datt 1998, 1999; Leuning et al. 2005; Ahl et al. 2006; Youngentob et al. 2012). Reflectance data were acquired from MODIS (Moderate Resolution Imaging Spectroradiometer) products MCD15A2, MCD43A4, MOD11A2, and MOD13A1, which were downloaded from NASA servers via the “MODIS” package in *R* at 8‐day intervals from 18 February 2000 to 22 September 2014 and processed to a spatial resolution of 500 m in Albers Equal Area Conic projection (Mattiuzzi 2014; R Core Team 2015). In addition to the commonly used Normalized Difference Vegetation Index (NDVI), several Eucalypt‐focused vegetation indices were constructed using the seven broad spectrum bi‐directionally adjusted Nadir BRDF reflectance bands from the MCD43A4 product. We based the Eucalypt‐focused indices on laboratory measurements performed by Datt (1998, 1999), which used narrow spectral bands measured from 118 Eucalypt leaf samples to identify indices of spectral reflectance wavelengths that were strongly correlated with chlorophyll *a* and *b*, carotenoids, and water content. From Datt's work (1998, 1999), we identified four indices that use reflectance wavelengths within the range of the seven broad reflectance bands from the MCD43A4 MODIS product, which allowed the construction of spatial variables that closely approximate these indices (Fig. S2 and Table S1).

Climate variables summarizing cumulative precipitation and temperature anomaly were developed, covering the same time period and 8‐day intervals as the MODIS data. Previously published studies, published interviews with apiarists, and preliminary data analysis, all suggest that climatic conditions experienced by Eucalypt forests during important phenological phases, such as temperature during bud production and water availability in the past 1–2 seasons, drive flowering and nectar production in Eucalypts (Moncur 1992; Williams and Woinarski 1997; Birtchnell and Gibson 2008; Hudson et al. 2009; Rawal et al. 2014a). Hence, climate data were structured to reflect climate extrema over relevant time lags. Raw climate data (daily rainfall and minimum and maximum temperature grids; Australian Bureau of Meteorology) were resampled using the nearest neighbor method to 500 m, matching the spatial resolution of the MODIS data. Precipitation values were summed over time, giving cumulative precipitation for the preceding 1, 3, 6, 9, 12, 15, and 18 months. Temperature anomaly values were calculated by comparing observed value of each grid cell for a given day to the mean value for the same day each year during the 14 year span of the data. Cumulative anomalies over multiple temporal intervals were calculated by adding all anomaly values for a grid cell for the preceding 1, 3, 6, 9, 12, 15, and 18 months.

In all, we developed 83 variables including unique Eucalypt‐based vegetation indices, temperature anomalies, and cumulative precipitation with various time‐lagged differences to capture long‐ and short‐term temporal changes. A full list of all variables used in the analysis can be found in Table S2.

### Dataset construction using a spatial sampling algorithm

Prior to modeling, datasets relating bat population response to spatial predictors were constructed. Bats are central place foragers, where they roost colonially and commute to foraging patches over a broad landscape (Elliott 1988). Although this type of foraging behavior facilitates population counts at roosts, the aspects of the environment that determine presence and abundance are found at foraging sites distributed across the surrounding landscape. A spatial sampling algorithm sampled the area surrounding a roost subject to spatial constraints based on previous knowledge of bat foraging behavior. First, sampling was restricted to areas containing preferred diet plant species of fruit bats (Eby and Law (2008), delineated by selecting polygons from regional ecosystem maps (produced by the Queensland Herbarium) that represent vegetation groups containing these diet species (Neldner 2004). Second, within the restricted sampling area, the weighted mean was taken using a spatial kernel representing the distribution of fruit bat foraging activity. The spatial kernel was defined by a Gompertz probability density function (using the “flexsurv” package in R; Jackson 2014) given by:

$$f\left(x|a,b\right)=be^{ax}e^{-\frac{b}{ae^{ax}}-1}$$

**Figure 2:**
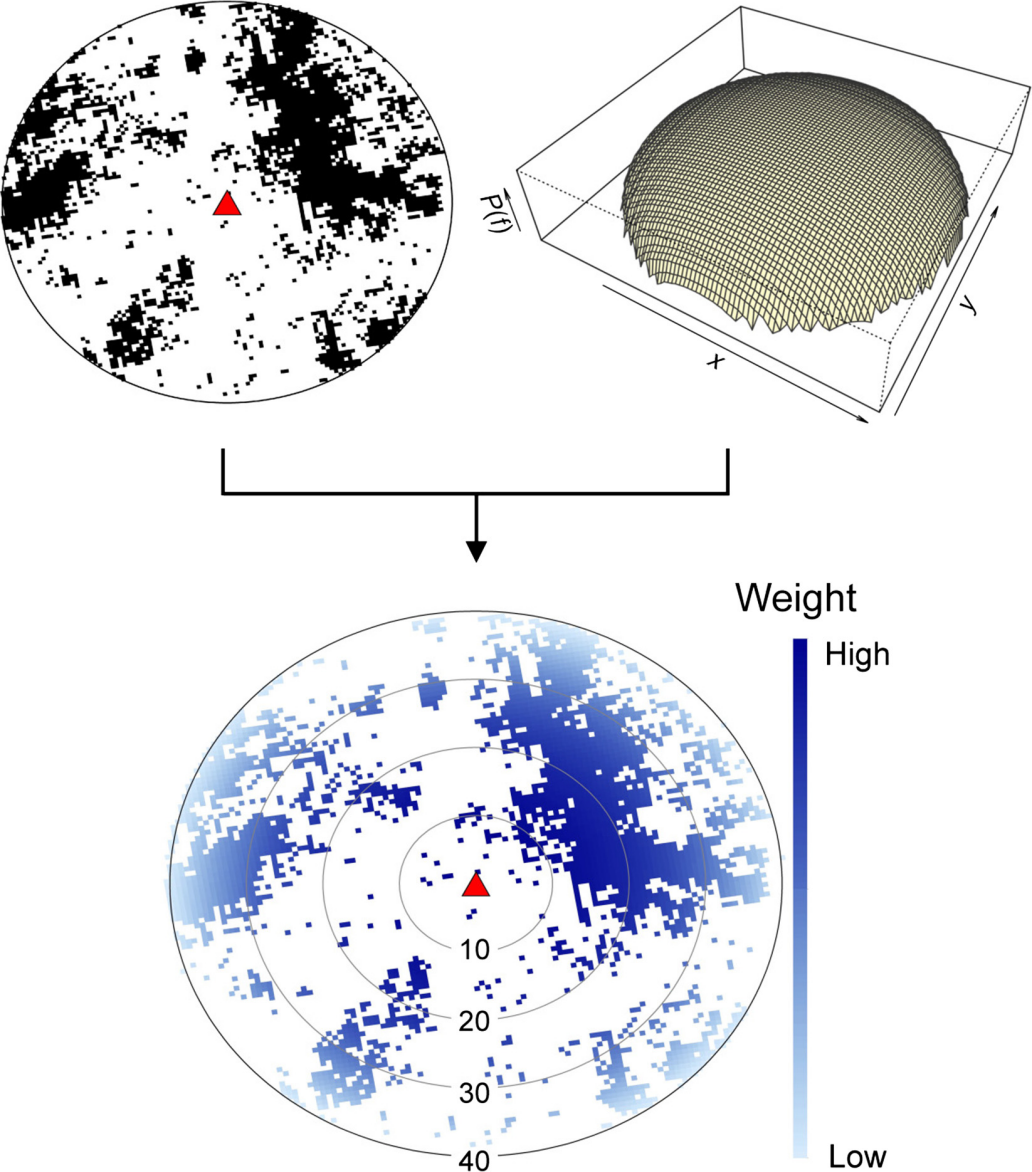
Schematic of the spatial sampling algorithm, which takes a binary raster layer depicting areas containing diet plant species (circle with black pixels), and weights them according to a spatial kernel defined by a probability density function (perspective plot). The result is a spatially weighted sample within a constrained area (bottom circle with blue pixels showing the sampling weight).

Bat telemetry studies have observed typical foraging distances within 20 km, with maximum commutes of 30–56 km (Palmer and Woinarski 1999; Roberts 2012). Hence, the decay rate of the Gompertz function was adjusted using the scale (*a *= 0.6) and rate (*b *= 1) parameters so that pixels within 20 km are strongly weighted with exponential decay between 20 and 40 km, creating an inverted bowl shape with steep sides centered on the roost (Fig. 2). The spatially weighted mean was calculated for rasters giving a time series with regular intervals for each spatial variable. The values at the time of census counts were calculated using linear interpolation between the two nearest time steps. The algorithm produces spatially and temporally relevant datasets of predictors associated with each census count (for model building), and a time series of each variable for the study roosts (for model projection).

### Statistical learning and model selection

Generalized linear models were fitted, with binomial errors to model occurrence (absence was recorded for population counts below 1000), and negative binomial errors (with a log link function) for abundance of bats, implemented with the “MASS” package in R (Venables and Ripley 2002). An absence threshold of 1000 was used because some roosts may be occupied by a few hundred sedentary bats that do not relocate in response to Eucalypt phenology (during preliminary analyses, model predictions held given thresholds between 500 and 2000 bats). In all three census datasets, the variance in count data was larger than the mean (Sandgate: *n* = 79, $\overset{¯}{x}$ = 2.4, *s* ^2^ = 4, Canungra: *n* = 81, $\overset{¯}{x}$ = 6.3, *s* ^2^ = 23.1, and Lowood: *n* = 85, $\overset{¯}{x}$ = 2.7, *s* ^2^ = 6.7), indicating overdispersion, and necessitating the use of the negative binomial distribution. An exhaustive search of all combinations of up to five predictors was performed for both binomial and negative binomial models. The five predictor limit was chosen because preliminary analysis using a forward‐stepwise selection algorithm indicated overfitting beyond 5. Models with any predictors correlated above a Pearson's correlation coefficient of |0.7| were removed prior to model fitting and selection criteria (Dormann et al. 2013). Models with the lowest AIC for each model size were retained, giving five candidate models in the best subset. Cross‐validated test error was then calculated for each model, and the most parsimonious model within one standard error of the lowest estimated test error was selected. Given the high amount of variability in the census counts, 95% bootstrapped confidence intervals were calculated with 1000 replications (using the “boot” package in R; Canty and Ripley 2015) to quantify the uncertainty in selected models.

A cross‐validation technique was designed that alleviates time dependency issues with time series data, but still utilizes a large portion of the data to calculate a robust test error measurement. Based on recommendations from Hastie et al. (2001), each dataset was split into two sets prior to model selection – one for model selection and the other for final model assessment (James et al. 2013). Specifically, 20% of observations at the end of each time series were withheld for final model assessment (the “test set”) and the preceding 80% comprises the “selection set”. Within the selection set, 50% of observations were allocated to model training, 20% to cross‐validation (the “validation set”), and the remaining 10% allows for multiple replications of cross validation using a rolling window method which performs forecasts and backcasts within the selection set (Arlot and Celisse 2010; Bergmeir and Ben?tez 2012). Within the test set and validation sets, the first 5 points are excluded to limit the confounding effect of temporal autocorrelation when estimating prediction error (Bergmeir and Benítez 2012). The mean log loss (MLL) and the root mean squared log error (RMSLE) were used to estimate prediction error for binomial and negative binomial models respectively (using the “Metrics” package in R; Hamner 2012).

Final models were selected by applying the one standard error rule on cross‐validation error metrics, which states that any model from the cross‐validation curve can be chosen provided it is within one standard error of the minimum error (Hastie et al. 2001; James et al. 2013). Final models were checked for over‐fitting by performing forecasts using a 50/50 training/testing split of the data for the Lowood and Canungra roosts, and a 60/40 split for Sandgate (due to smaller sample size). We calculated MLL and classification accuracy of forecasted Binomial models, and RMSLE for forecasted Negative Binomial models. After confirming that selected models were not overfitting the training data, they were refitted using all observations. When refitting to all observations, we investigated generalized additive models (GAMs) with smoothed spline terms for each predictor in the model to accommodate any nonlinear relationships between predictors and response. Incidence of Hendra virus infection in horses and humans occurring within the 40 km foraging radius of each roost was overlaid on the time series to compare bat population flux due to Eucalypt nectar phenology and timing of Hendra virus spillover incidence.

## Results

### Proxy variables

Spatial database development and dataset building via the spatial sampling algorithm described above produced three datasets with bat population response and a suite of spatial variables to be mined for optimal predictor combinations. The spatial variables and their time‐lagged differences show variable levels of correlation with bat population response in all three datasets (Figs. S3–S5). Correlation patterns are different in each dataset, reflecting the unique species assemblages surrounding each roost. Datasets for Canungra and Sandgate showed highest correlations for variables depicting time‐lagged changes in ECARR (Eucalypt chlorophyll *a* reflectance ratio) and average minimum and maximum temperature, with several temporal intervals exhibiting correlation coefficients >|0.5|. The dataset for Lowood shows high correlation with ECARR, EWDI2 (Eucalypt wetness difference index 2), PREC_12mo, PREC_18mo (cumulative precipitation of the past 12 and 18 months), MSI (moisture stress index), and NDVI_9mo (change in normalized difference vegetation index over 9 months).

### Model selection

**Figure 3:**
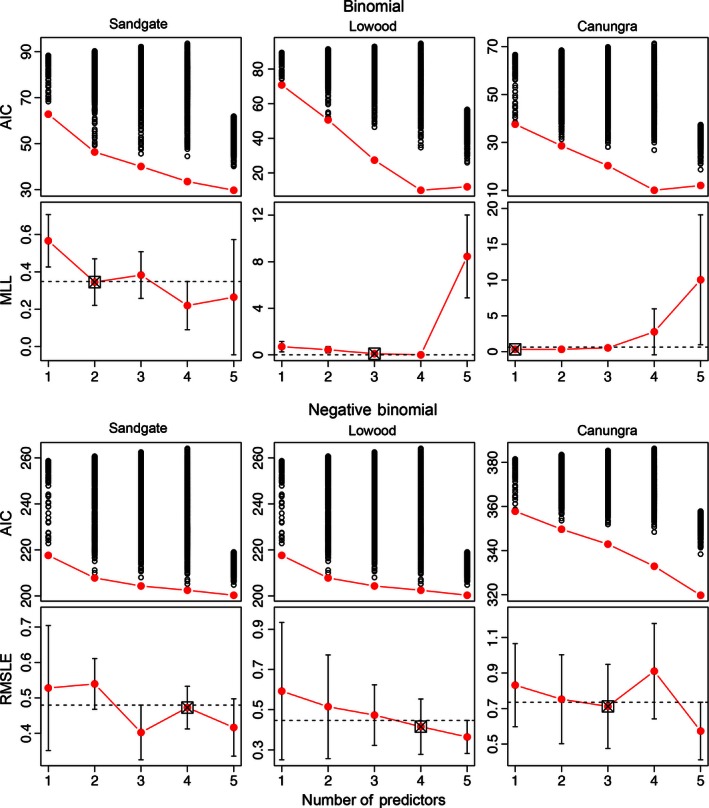
Results of the exhaustive model search (top panels giving the AIC) and cross‐validated test error of the subset of best models [bottom panels giving the mean log loss (MLL) and the root mean squared log error (RMSLE)]. The dotted line is the maximum standard error value of the model with the minimum test error. Models below this line may be selected as the best model. Selected models are indicated with boxes.

Our statistical learning approach searched through all models with up to five uncorrelated parameters, and yielded Binomial and Negative Binomial models capable of predicting occurrence and abundance of bat populations at all three roosts. Final models were selected based on performance on a limited set of training data, then validated by comparing model predictions with independent test data. Figure 3 shows results of the model selection process, with the distribution of AIC scores for all fitted models and the cross‐validated test error for the best performing model of each size. Selected models had a minimum model size of 1 and a maximum of 4 across all models (Fig. 3). We allowed two concessions to the one standard error rule. First, the four‐predictor Binomial model for the Lowood roost attained complete separation of the response, giving it the lowest cross‐validation error on the training data (MLL = 0; Table 1), so we selected the three‐parameter model instead. For the Sandgate roost, the three‐, four‐, and five‐parameter Negative Binomial models were within one standard error of the minimum cross‐validated test error (Fig. 3 and Table 2). We chose the four‐parameter model even though it was not the most parsimonious model, based on its superior performance on the independent test data withheld from the model selection procedure (RMSLE = 0.67 vs. 0.72).

**Table 1:** Best performing binomial models of each size with selected models in bold. See Table S2 for detailed descriptions of variables

Binomial				
	Model	AIC	MLL	SE
Sandgate	Occurrence ~ ANOMtmn_18mo	62.79	0.57	0.14
	**Occurrence ˜ ECBRR_3mo + ANOMtmn_18mo**	**46.36**	**0.35**	**0.12**
	Occurrence ~ ECBRR_9mo + ANOMtmn_18mo + AVGtmx_1mo_15mo	40.05	0.38	0.13
	Occurrence ~ ECARR_9mo + ANOMtmn_18mo + PREC_9mo + PREC_18mo	33.49	0.22	0.13
	Occurrence ~ MSI_9mo + ANOMtmn_18mo + AVGtmx_1mo_15mo + PREC_9mo + PREC_18mo	29.70	0.26	0.31
Lowood	Occurrence ~ EWDI1_9mo	70.78	0.70	0.45
	Occurrence ~ ECARR + EWDI1_12mo	50.65	0.44	0.27
	**Occurrence ˜ ECARR + EWDI1_12mo + NDVI_3mo**	**27.38**	**0.10**	**0.07**
	Occurrence ~ ECBRR + ECBRR_3mo + EWDI1_9mo + EWDI1_18mo	10.00	0.00	0.00
	Occurrence ~ ECARR + ECARR_9mo + EWDI1_9mo + MSI_12mo + AVGtmx_1mo_9mo	12.00	8.46	3.56
Canungra	**Occurrence ˜ ECARR_18mo**	**37.56**	**0.31**	**0.24**
	Occurrence ~ NDVI_18mo + AVGtmn_1mo_3mo	28.57	0.31	0.31
	Occurrence ~ EWDI1_12mo + NDVI_18mo + AVGtmn_1mo_3mo	20.25	0.51	0.28
	Occurrence ~ ECBRR_18mo + ANOMtmn_3mo + AVGtmx_1mo_15mo + PREC_3mo	10.00	2.75	3.21
	Occurrence ~ ECBRR_18mo + ANOMtmn_3mo + ANOMtmx_3mo + AVGtmx_1mo_3mo + PREC_3mo	12.00	10.02	9.08

**Table 2:** Best performing negative binomial models of each size with selected models in bold. See Table S2 for detailed descriptions of variables

Negative Binomial				
	Model	AIC	RMSLE	SE
Sandgate	Abundance ~ AVGtmn_1mo	217.63	3.58	0.73
	Abundance ~ ANOMtmn_18mo + AVGtmn_1mo	207.85	3.74	1.13
	Abundance ~ ECARR + EWDI1_15mo + ANOMtmn_18mo	204.34	2.49	0.51
	**Abundance ˜ ECARR_6mo + MSI_15mo + ANOMtmn_12mo + ANOMtmn_18mo**	**202.50**	**5.17**	**3.20**
	Abundance ~ ECBRR_9mo + EWDI1_15mo + NDVI_9mo + ANOMtmn_18mo + AVGtmx_1mo_3mo	200.31	1.82	0.90
Lowood	Abundance ~ EWDI1_18mo	269.01	0.59	0.34
	Abundance ~ ECARR + ECBRR_18mo	250.04	0.51	0.26
	Abundance ~ ECBRR + EWDI1_18mo + EWDI2_3mo	239.96	0.47	0.15
	**Abundance ˜ ECBRR + EWDI1_18mo + NDVI_3mo + ANOMtmx_9mo**	**230.72**	**0.42**	**0.14**
	Abundance ~ ECARR + ECBRR_18mo + EWDI1_12mo + EWDI2_3mo + NDVI_18mo	222.90	0.36	0.08
Canungra	Abundance ~ AVGtmx_1mo	357.85	0.83	0.23
	Abundance ~ ANOMtmn_18mo + AVGtmx_1mo	349.68	0.75	0.25
	**Abundance ˜ ANOMtmn_18mo + AVGtmn_1mo_9mo + AVGtmx_1mo_3mo**	**342.92**	**0.71**	**0.24**
	Abundance ~ EWDI1_6mo + AVGtmx_1mo_3mo + PREC_3mo + PREC_15mo	332.89	0.91	0.27
	Abundance ~ EWDI1_6mo + ANOMtmx_18mo + AVGtmx_1mo_3mo + PREC_3mo + PREC_18mo	319.73	0.57	0.16

**Figure 4:**
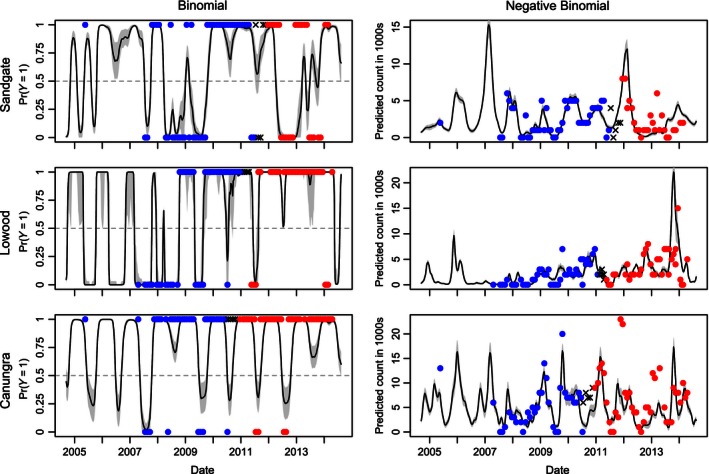
Predictive performance of final selected models with a 50/50 training/testing split. Data points from the training set are displayed in blue, and data points from the test set are displayed in red with the points in the temporal spacer displayed as black exes. 95% bootstrapped confidence intervals are shown in gray.

We confirmed that selected models were not overfitting the data by projecting fitted values onto independent test data with a 50/50 split (Fig. 4). Binomial models predicted roost occupancy of the independent test data with an average 86% accuracy (Sandgate = 73%, MLL = 0.82, Lowood: 90%, MLL = 1.17, Canungra: 95%, MLL = 0.24) and Negative Binomial models predict patterns of abundance congruent with independent test data (Sandgate: Pseudo *R* ^2^ = 0.66, RMSLE = 0.66, Lowood: Pseudo *R* ^2^ = 0.56, RMSLE = 0.59, Canungra: Pseudo *R* ^2^ = 0.62, RMSLE = 0.67). Performance of models built with 50% of the total data should not be over‐interpreted. Model forecasts (Fig. 4) and performance metrics at this point are meant to illustrate that the models are not over‐fitting the data, and they are not meant to show that models can forecast future population flux.

### Model performance

**Figure 5:**
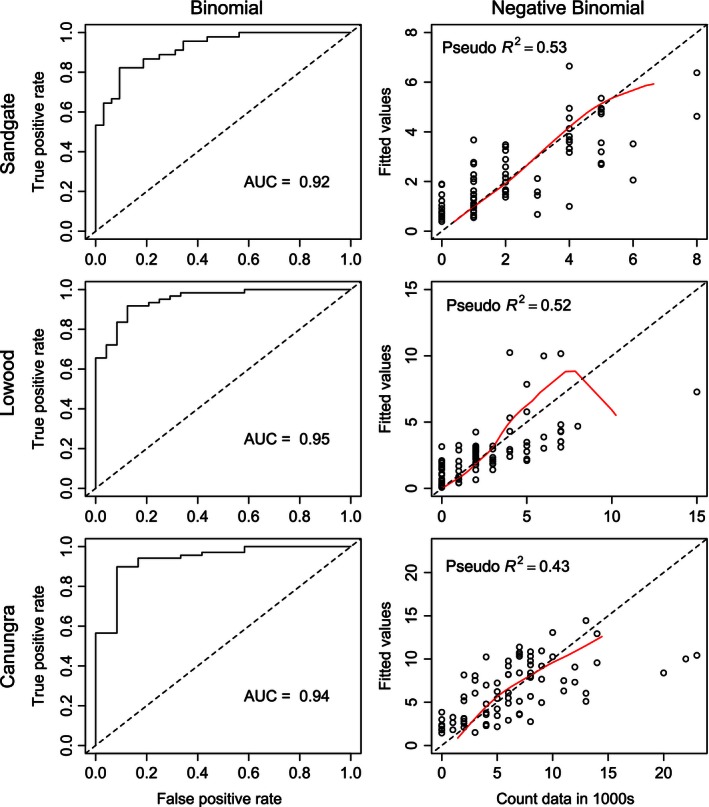
Model performance metrics of final models fitted to all data. ROC curves and AUC shown on the left, and plots of fitted versus data values with Pseudo *R* ^2^ on the right. For additional model performance metrics, such as thresholds used to calculate classification accuracy and scaled residual plots, see Fig. S6.

Final models chosen by the model selection procedure exhibit adequate fit when refitted with all observations. Both Binomial and Negative Binomial GAMs with smoothed spline terms for each predictor returned essentially the same models with marginal improvement in fit. Binomial models achieved an average AUC score of 0.91 and classification rate (CR) of 0.9 (Sandgate: AUC = 0.92, CR = 0.86, Lowood: AUC = 0.95, CR = 0.91, Canungra: AUC = 0.94, CR = 0.93), and Negative Binomial models achieved an average Pseudo *R* ^2^ value of 0.49 (Sandgate = 0.53, Lowood = 0.52, Canungra = 0.43; Fig. 5 and Fig. S6). Generally, Binomial models predicted roost occupancy using fewer predictors, the majority of which were remotely sensed variables, potentially because detection of physical changes in the tree canopy is sufficient. In the case of Canungra, occupancy can be predicted with only one parameter, ECARR_18mo (change in chlorophyll *a* reflectance ratio over 18 months). Overall, models predicting abundance included climate variables as well (Table 1). This is likely attributable to variable nectar production during flowering events; climate variables may be a better indicator of the magnitude of nectar production (Birtchnell and Gibson 2008).

### Predicted population flux and spillover

**Figure 6:**
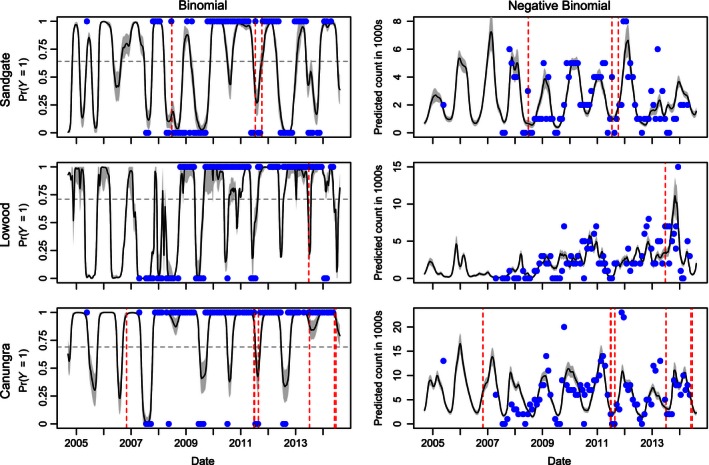
Final models of bat population flux using all data points with the dates of spillover events that occurred within a 40 km foraging radius of each roost respectively shown as dotted red lines. 95% bootstrapped confidence intervals are shown in gray.

Comparison of spillover incidents and models of population abundance reveal that spillover events, which historically occur during winter (July–October) in the subtropics (Plowright et al. 2015), tend to occur in winter when proxies of nectar abundance predict the absence of large aggregations of bats (Fig. 6). Specifically, Binomial models of roost occupancy show the four spillover events that occurred near Sandgate and Lowood occurred at times the two roosts were occupied (>1000 bats), but predicted to be unoccupied based on nectar‐based resources. Conversely, 7 of the 8 spillover events near Canungra occurred when models predicted high probability of occupancy. However, maximum population counts were higher at Canungra and presence of at least 1000 individuals was almost continuous. Models of population abundance predict a mean of 3020 bats at the time of observed spillover events, which is relatively low considering the large aggregations commonly observed with fruit bats (Eby 1991; Shilton et al. 2008; Roberts et al. 2012a).

## Discussion

Our models demonstrate a mechanistic link between spatial proxies of nectar phenology and bat population flux. To our knowledge, this is the first study to accurately model the occurrence of fruit bats at day roosts and explain a significant portion of the variation in abundance over time. Previous work has modeled the spatial extent of bat habitat or potential areas where Hendra virus spillover could occur, based on bat roost locations, regardless of occupancy (Parsons et al. 2010; Hahn et al. 2014a,b; Smith et al. 2014; Thanapongtharm et al. 2015). Our methods show how models of bat population flux at the roost scale can be built, facilitating landscape‐scale spatiotemporal predictions of foraging suitability and bat population distribution. The success of our models (measured by their ability to classify occupancy with ~90% accuracy and explain ~50% of the variability of abundance) stems from a well‐informed suite of proxy variables, a sampling algorithm that relates predictors to population response in an ecologically relevant manner, and a statistical learning algorithm that identifies optimal predictor sets. Models relied most on time‐lagged changes of Eucalypt‐focused vegetation indices (which likely detect structural changes in the forest canopy as Eucalypts undergo phenological cycles of growth and flowering (Zhang et al. 2003; Ahl et al. 2006) and temperature extrema (which trigger synchronized flowering events within species; Moncur 1992; Rawal et al. 2015), indicating that temporal changes in bat population abundance at these roosts are largely driven by cycles of nectar production in the surrounding landscape. Comparison of spillover to models of occupancy and abundance suggests that (1) spillover near our study roosts occurred when nectar‐based resources are predicted to support relatively low population abundance, and (2) the relationship between spillover risk and population abundance may be heterogeneous across the region. This is the first time that spillover has been related to temporal trends in bat population flux driven by nectar foraging suitability, and given the limited data on spillover and high degree of stochasticity in bat disease ecology, further analyses are required.

General limitations of our study are worth consideration. First, our analysis focuses on three study roosts because they contain census time series with sufficient sample sizes for robust temporal cross‐validation, and spillover events have occurred within their foraging radii, allowing comparison of population flux and spillover. Although many other roosts are found in southeast Queensland, we expect that they exhibit similar seasonal patterns of occupancy based on their proximity to our study roosts. The observed patterns at the three roosts – spillover occurring in winter during low predicted population sizes – is corroborated by studies showing that viral excretion (Field et al. 2015b) and spillover (Plowright et al. 2015) in the subtropics are highest in winter, coincident with smaller winter population sizes (Nelson 1965; Eby 1991). Second, the set of variables which predict bat occurrence and abundance is unique between roosts. This is expected because the assemblage of Eucalypt species – each with a species‐specific flowering response to climate – surrounding each roost is unique (Keatley and Hudson 2007; Birtchnell and Gibson 2008), necessitating an individual model fitted to each roost data set. However, specific predictor–response relationships at the roost scale do not preclude developing landscape‐scale models, as discussed below. Third, models detect the pattern of occupancy with high classification accuracy (86–93%); however, predicting bat abundance is more challenging, with 43–53% of the variation in the data accounted for. Given the overdispersion in the abundance data (due to variable population response and human error in estimating census counts) coupled with error/approximation associated with climate and remote sensing variables, accounting for half the variation in bat population response is noteworthy. It should also be noted that foraging behavior in *P. alecto* and *P. poliocephalus* is flexible. They primarily feed on nectar and pollen of Eucalypts (Parry‐Jones and Augee 1991; Courts 1998); however, when these are unavailable, they can remain at a roost by switching to non‐nectar‐based resources such as fruits of non‐native plants found in urban and peri‐urban landscapes (Eby 1998; Markus and Hall 2004; Fahr et al. 2015; Field et al. 2015a). The extent to which populations utilize, and are able to be supported by, urban and peri‐urban areas is unknown. However, generalist feeding behavior could explain occurrence or high abundance at times when models based on nectar phenology predict absence or low abundance. Furthermore, social drivers of colonial behavior in bats can lead to high peaks in abundance that are not necessarily driven by food abundance (nectar‐based or otherwise).

Our models provide an important observation related to the temporal relationship between pathogen spillover and bat population dynamics due to nectar foraging; that is, Hendra virus spillover in this region is likely not driven by large groups of migratory bats that feed on nectar and pollen of Eucalypts. Most Hendra virus spillover events occur in winter in southeast Queensland when our abundance models predict relatively low (mean of 3020) population sizes at roosts. Interpretation of spillover risk based on our models and data alone is speculative; however, they support the hypothesis that spillover may be associated with resident sedentary subpopulations that choose to remain in the roost and subsist on urban‐based resources when nectar availability is low (Plowright et al. 2015). General foraging behavior of fruit bats allows them to form sedentary populations in response to both seasonal changes in nectar availability and loss of foraging habitat. If winter population sizes approach or surpass the seasonal carrying capacity of the landscape, and food resources are limited, part of the population may also suffer from nutritional stress. Further, lower night time temperatures may increase caloric requirement and the cost of immune functioning (Hawley and Altizer 2011), facilitating winter nutritional stress, which Plowright et al. (2015) propose may lead to increased viral shedding. Although physiological stress is a recurrent theme in disease ecology (Hawley and Altizer 2011; Brearley et al. 2013; Hing et al. 2016) and has been observed in the Hendra virus disease system (Plowright et al. 2008), its role in Hendra virus spillover is difficult to determine without additional data on chronic or periodic states of stress in bats (McMichael et al. 2014). Alternatively, increased urban foraging in winter, leading to increased contact with horses, could be the only risk factor needed to drive spillover.

Proxies of nectar availability can explain half the variation in bat population flux observed over a 10‐year period (2004–2014), which straightforwardly suggests that approximately 50% of the bat population flux is driven by nectar‐based resources; however, this may be an over‐simplification. Parry‐Jones and Augee (1991) observed remarkable variability in *P. poliocephalus* in the proportion of diet comprised of nectar and pollen of Eucalypts (28–86% depending on the year and season). Therefore, we hypothesize long‐term shifts toward a more urban‐based diet with less migration in bat foraging behavior as foraging habitat becomes fragmented and the cost of migrating for a nectar dominated diet increases. Dramatic landscape changes in southeast Queensland began to accelerate in the early 1990s (just prior to Hendra virus emergence in 1994 (Bradshaw 2012). Additionally, modeling studies suggest Eucalypts may respond to climate changes with increased asynchrony and decreased intensity of flowering events (Keatley et al. 2002; Hudson et al. 2009, 2011a; Butt et al. 2013; Rawal et al. 2013, 2014a,b, 2015). The combined effect of habitat fragmentation and climate changes may be decreased abundance, increased spatial heterogeneity, and increased asynchrony of nectar resources. Together, these processes may decrease the overall amount of energy available across the landscape and increase the energetic cost of a mobile foraging strategy based primarily on nectar resources (Calcagno et al. 2014). It is plausible that the past 25 years of landscape and climate changes in southeast Queensland have altered the ecological behavior of nectar‐seeking in fruit bats leading to supplementation with non‐nectar resources in peri‐urban areas, consequently diminishing the ability of models based on nectar resources to explain bat abundance.

The tools we have developed for modeling bat population abundance at day roosts is an important first step to understand spatiotemporal dynamics of existing nectar resources. *Pteropus alecto* and *Pteropus poliocephalus* rely on large population sizes to search out patchy and ephemeral pulses of nectar resources, making it difficult to define discrete spatial limits of suitable habitat (Eby et al. 1999). Techniques to estimate the distribution of nectar‐based resources are important, because they can inform about habitat loss over time, potential seasonal nectar shortages, and times and areas when bat populations are likely to rely more on urban‐based resources leading to increased risk of spillover. Expanding models to the landscape scale could be achieved with the same spatial sampling techniques to construct datasets for additional locations, but would use regression trees, which allow multiple predictor–response relationships within datasets, thereby permitting prediction of nectar‐driven population density across space and time. This can direct better‐informed policy decisions concerning the conservation of bat foraging habitat, which has become a pressing question as humans and bats increasingly compete for habitat.

The results presented here demonstrate that indices of Eucalypt leaf physiology can be approximated with spectral reflectance data, and when combined with climate variables representing anomalies over time, they are effective predictors of fruit bat population flux. Building on the tools here, further modeling studies can address more complex questions such as individual species responses and spatiotemporal patterns of resource distribution. Future work will enable identification of habitat areas of high conservation priority that consider the complexities of bat foraging ecology. These can be extended to other ecological systems where fruit bat populations are driven by food resource phenology, such as African fruit bats, which migrate among Miombo forests (Richter and Cumming 2006), and Asian fruit bats that follow the phenology of Durian fruit and petai bean (Epstein et al. 2009). We consider this an important area of research as bat population distribution, environmental changes, and human behavior are inseparable from disease dynamics and mitigating risk of spillover to humans (Wood et al. 2012).

## Conflict of Interest

None declared.

## Supporting information


**Figure S1.** Distribution of the four fruit bat species in Australia and the locations of observed spillover events.
**Figure S2.** A typical reectance curve for Eucalypts and placement of the seven reectance bands of the MCD43A4 MODIS product.
**Figure S3.** Correlation (Pearsons r) between 83 spatial variables and the log total population count at the Sandgate roost.
**Figure S4.** Correlation (Pearsons r) between 83 spatial variables and the log total population count at the Lowood roost.
**Figure S5.** Correlation (Pearsons r) between 83 spatial variables and the log total population count at the Canungra roost.
**Figure S6.** Model performance metrics of final models fitted to all data. The columns from left to right display: AUC, threshold of maximum classification accuracy, plots of fitted versus data values with Pseudo R2, and residuals versus fitted values.
**Table S1.** Descriptions and equations for commonly used vegetation indices (NDVI and MSI), and four Eucalypt‐specific vegetation indices (ECARR, ECBRR, EWDI1, EWDI2) from Datt (1998, 1999), which were approximated using broad spectrum reflectance bands of the MCD43A4 MODIS product.
**Table S2.** A complete list of the 83 spatial variables and their time‐lagged differences.
