# Supplementary material for: Models of Eucalypt phenology predict bat population flux
Source: Ecol Evol. 2016 Sep 21;6(20):7230–45. doi: 10.1002/ece3.2382 (PMC5115174; doi:10.1002/ece3.2382)
Supplement: Supplementary file 1 — Figure S1. Distribution of the four fruit bat species in Australia and the locations of observed spillover events. Figure S2. A typical reectance curve for Eucalypts and placement of the seven reectance bands of the MCD43A4 MODIS product. Figure S3. Correlation (Pearsons r) between 83 spatial variables and the log total population count at the Sandgate roost. Figure S4. Correlation (Pearsons r) between 83 spatial variables and the log total population count at the Lowood roost. Figure S5. Correlation (Pearsons r) between 83 spatial variables and the log total population count at the Canungra roost. Figure S6. Model performance metrics of final models fitted to all data. The columns from left to right display: AUC, threshold of maximum classification accuracy, plots of fitted versus data values with Pseudo R2, and residuals versus fitted values. Table S1. Descriptions and equations for commonly used vegetation indices (NDVI and MSI), and four Eucalypt‐specific vegetation indices (ECARR, ECBRR, EWDI1, EWDI2) from Datt (1998, 1999), which were approximated using broad spectrum reflectance bands of the MCD43A4 MODIS product. Table S2. A complete list of the 83 spatial variables and their time‐lagged differences. [file ECE3-6-7230-s001.pdf]

Supplementary Material

Models of Eucalypt phenology predict bat population flux

John R. Giles, Raina K. Plowright, Peggy Eby, Alison J. Peel, and Hamish McCallum

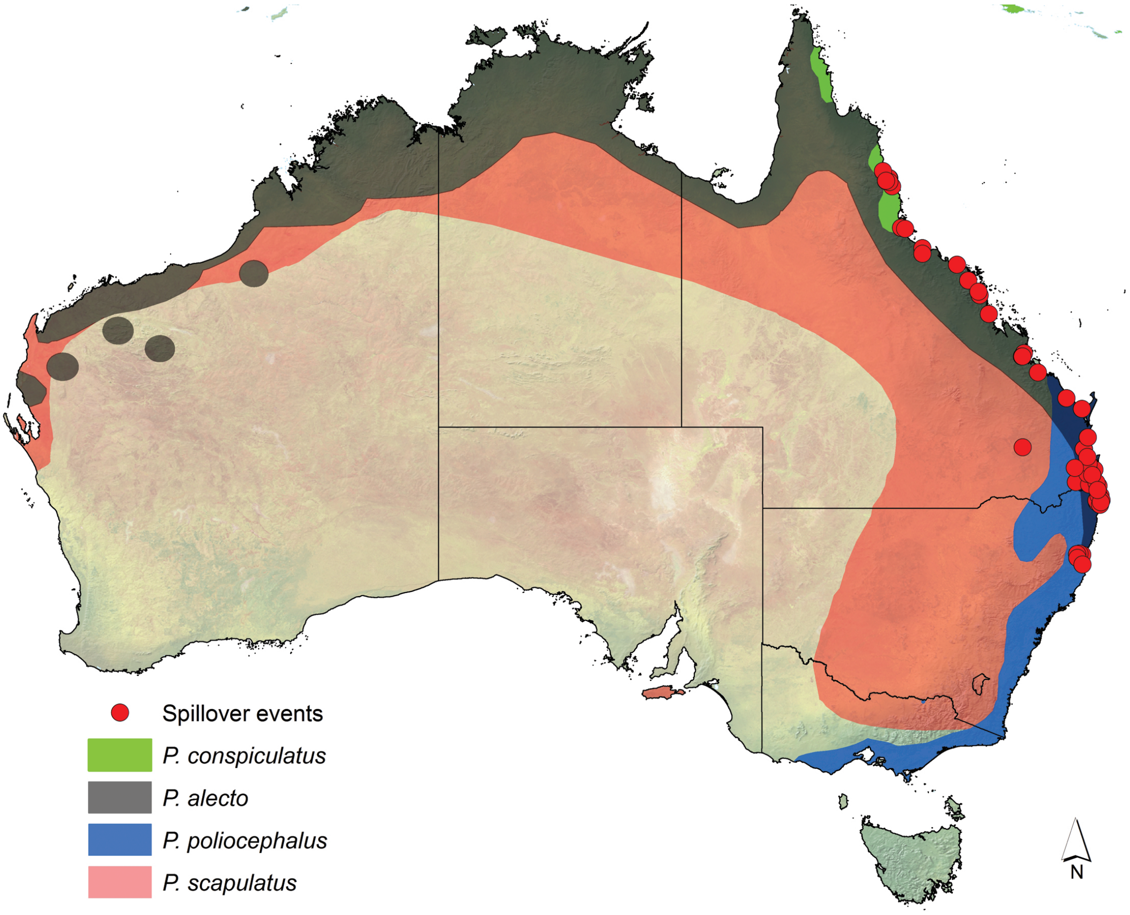

Figure A1: Distribution of the four fruit bat species in Australia and the locations of observed spillover events.

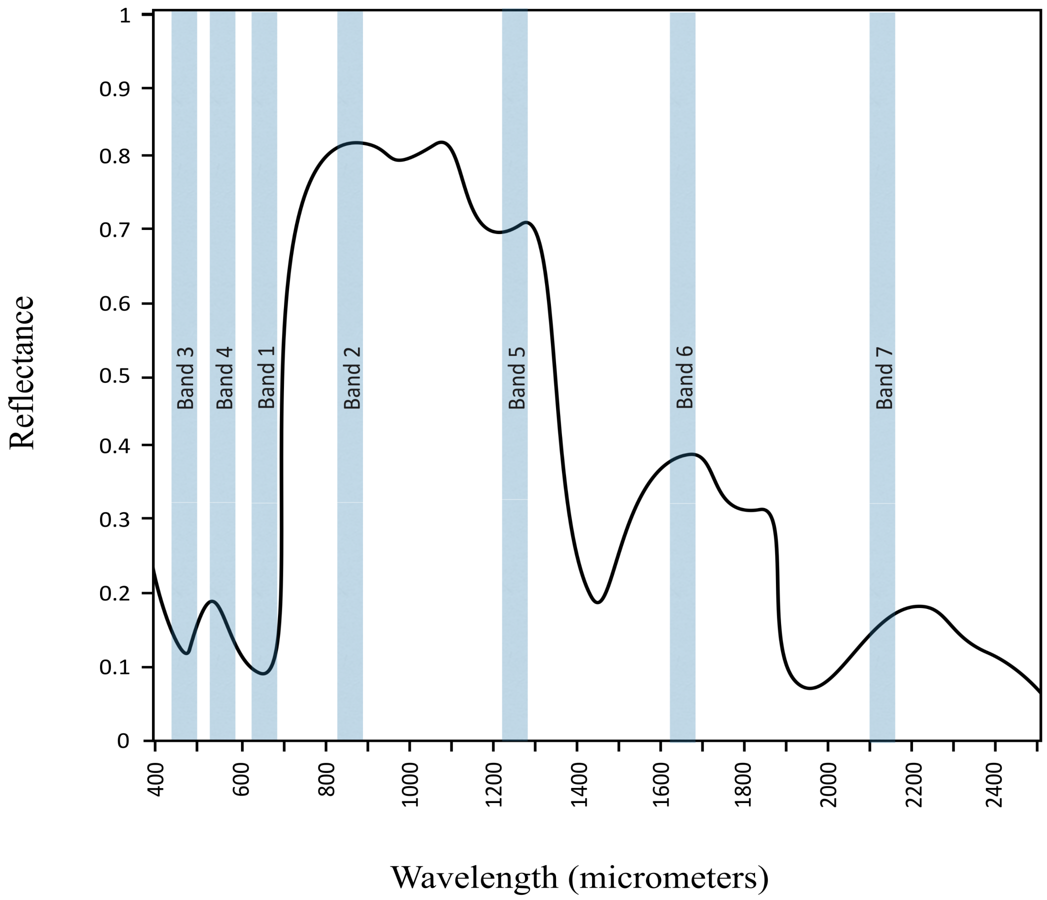

Figure A2: A typical reflectance curve for Eucalypts and placement of the seven reflectance bands of the MCD43A4 MODIS product.

Table A1: Descriptions of commonly used vegetation indices (NDVI and MSI), and four Eucalypt-specific vegetation indices (ECARR, ECBRR, EWDI1, EWDI2) from Datt (1998, 1999), which were approximated using broad spectrum reflectance bands of the MCD43A4 MODIS product.

Index	Description	Formula	Remotely sensed approximation
NDVI	Normalized Difference Vegetation Index	$(R_{NIR} - R_{Red})/(R_{NIR} + R_{Red})$	(Band 2 - Band 1)/(Band 2 + Band 1)
MSI	Moisture Stress index	R_{1650}/R_{820}	Band 6/Band 2
ECARR	Eucalypt Chlorophyll a reflectance ratio	$0.0161 \times [R_{672}/(R_{550} \times R_{708})]^{0.7784}$	$0.0161 \times [\text{Band2}/(\text{Band4} \times \text{Band1})]^{0.7784}$
ECBRR	Eucalypt Chlorophyll b reflectance ratio	$0.0337 \times (R_{672}/R_{550})^{1.8695}$	$0.0337 \times (\text{Band1}/\text{Band4})^{1.8695}$
EWDI1	Eucalypt Wetness Difference Index 1	$0.08 \times [(R_{850} - R_{2218})/(R_{850} - R_{1928})] - 0.052$	$0.08 \times [(\text{Band2} - \text{Band7})/(\text{Band2} - \text{Band6})] - 0.052$
EWDI2	Eucalypt Wetness Difference Index 2	$0.045 \times [(R_{850} - R_{1788})/(R_{850} - R_{1928})] - 0.014$	$0.045 \times [(\text{Band2} - \text{Band6})/(\text{Band2} - \text{Band7})] - 0.014$

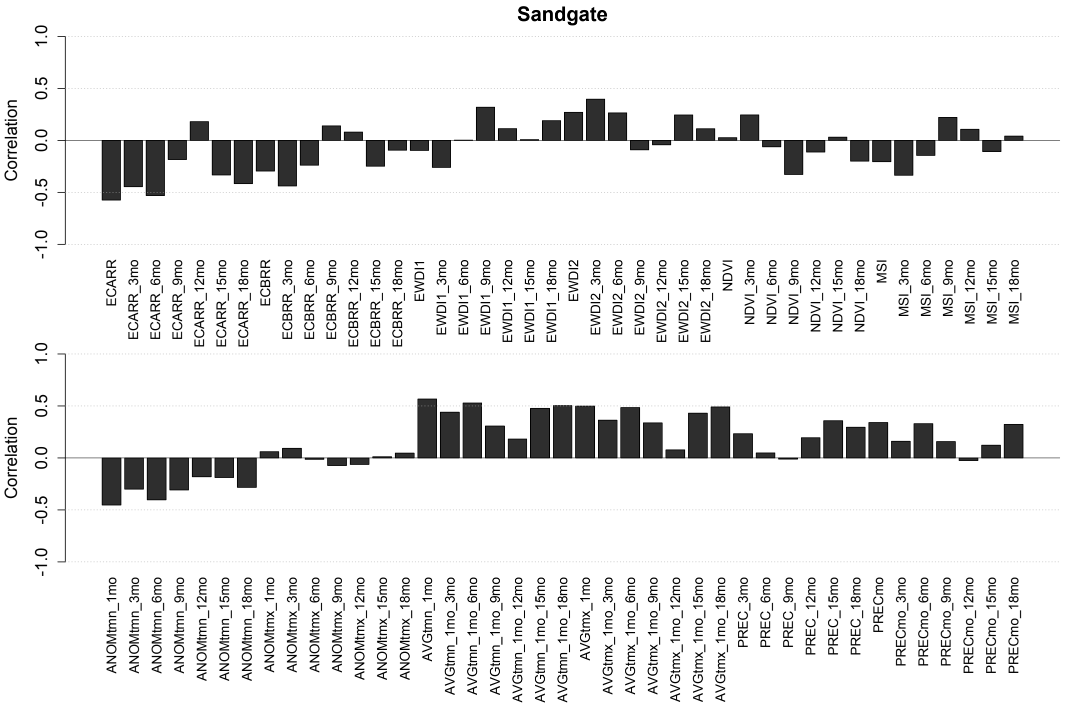

Figure A3: Correlation (Pearsons r) between 83 spatial variables and the log total population count at the Sandgate roost.

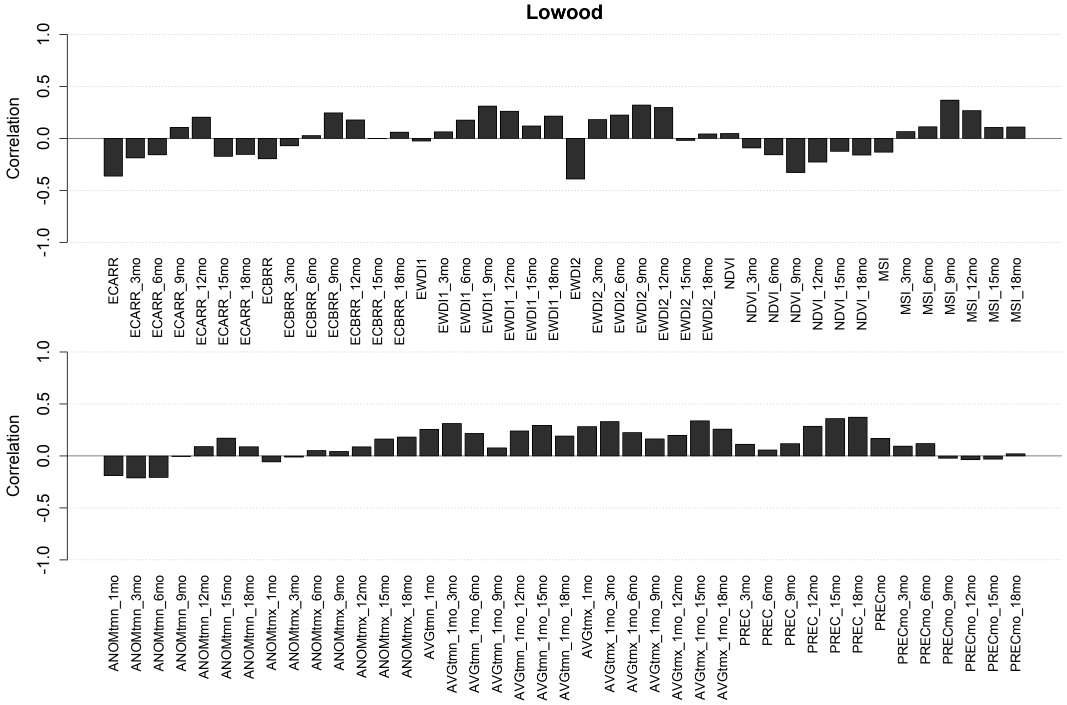

Figure A4: Correlation (Pearsons r) between 83 spatial variables and the log total population count at the Lowood roost.

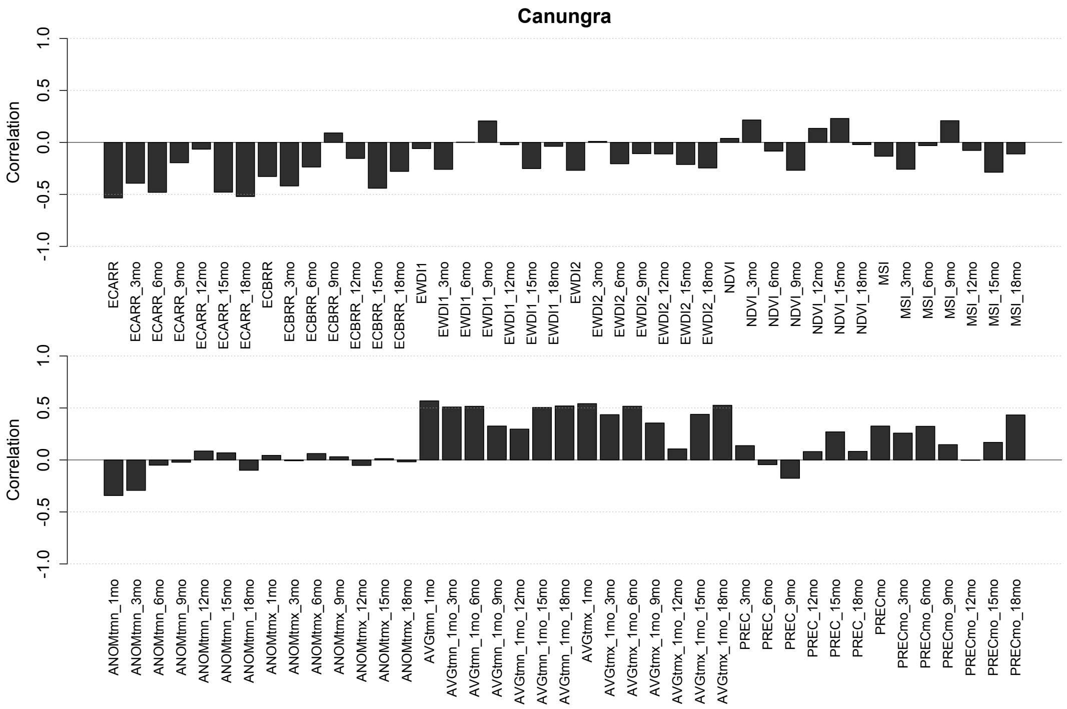

Figure A5: Correlation (Pearsons r) between 83 spatial variables and the log total population count at the Canungra roost.

Table A2: Descriptions of commonly used vegetation indices (NDVI and MSI), and four Eucalypt-specific vegetation indices (ECARR, ECBRR, EWDI1, EWDI2) from Datt (1998, 1999), which were approximated using broad spectrum reflectance bands of the MCD43A4 MODIS product.

Name	Description	Formula
ECARR	Eucalypt chlorophyll *a* reflectance ratio	$0.0161 \times [\text{Band2}/(\text{Band4} \times \text{Band1})]^{0.7784}$
ECARR_3mo	Change in ECARR over 3 months	$\text{ECARR}_t - \text{ECARR}_{t-3months}$
ECARR_6mo	Change in ECARR over 6 months	$\text{ECARR}_t - \text{ECARR}_{t-6months}$
ECARR_9mo	Change in ECARR over 9 months	$\text{ECARR}_t - \text{ECARR}_{t-9months}$
ECARR_12mo	Change in ECARR over 12 months	$\text{ECARR}_t - \text{ECARR}_{t-12months}$
ECARR_15mo	Change in ECARR over 15 months	$\text{ECARR}_t - \text{ECARR}_{t-15months}$
ECARR_18mo	Change in ECARR over 18 months	$\text{ECARR}_t - \text{ECARR}_{t-18months}$
ECBRR	Eucalypt chlorophyll *b* reflectance ratio	$0.0337 \times (\text{Band1}/\text{Band4})^{1.8695}$
ECBRR_3mo	Change in ECBRR over 3 months	$\text{ECBRR}_t - \text{ECBRR}_{t-3months}$
ECBRR_6mo	Change in ECBRR over 6 months	$\text{ECBRR}_t - \text{ECBRR}_{t-6months}$
ECBRR_9mo	Change in ECBRR over 9 months	$\text{ECBRR}_t - \text{ECBRR}_{t-9months}$
ECBRR_12mo	Change in ECBRR over 12 months	$\text{ECBRR}_t - \text{ECBRR}_{t-12months}$
ECBRR_15mo	Change in ECBRR over 15 months	$\text{ECBRR}_t - \text{ECBRR}_{t-15months}$
ECBRR_18mo	Change in ECBRR over 18 months	$\text{ECBRR}_t - \text{ECBRR}_{t-18months}$
EWDI1	Eucalypt wetness difference index 1	$0.08 \times [(\text{Band2} - \text{Band7})/(\text{Band2} - \text{Band6})] - 0.052$
EWDI1_3mo	Change in EWDI1 over 3 months	$\text{EWDI1}_t - \text{EWDI1}_{t-3months}$
EWDI1_6mo	Change in EWDI1 over 6 months	$\text{EWDI1}_t - \text{EWDI1}_{t-6months}$

EWDI1_9mo	Change in EWDI1 over 9 months	$\text{EWDI1}_t - \text{EWDI1}_{t-9months}$
EWDI1_12mo	Change in EWDI1 over 12 months	$\text{EWDI1}_t - \text{EWDI1}_{t-12months}$
EWDI1_15mo	Change in EWDI1 over 15 months	$\text{EWDI1}_t - \text{EWDI1}_{t-15months}$
EWDI1_18mo	Change in EWDI1 over 18 months	$\text{EWDI1}_t - \text{EWDI1}_{t-18months}$
EWDI2	Eucalypt wetness difference index 2	$0.045 \times [(\text{Band2} - \text{Band6})/(\text{Band2} - \text{Band7})] - 0.014$
EWDI2_3mo	Change in EWDI2 over 3 months	$\text{EWDI2}_t - \text{EWDI2}_{t-3months}$
EWDI2_6mo	Change in EWDI2 over 6 months	$\text{EWDI2}_t - \text{EWDI2}_{t-6months}$
EWDI2_9mo	Change in EWDI2 over 9 months	$\text{EWDI2}_t - \text{EWDI2}_{t-9months}$
EWDI2_12mo	Change in EWDI2 over 12 months	$\text{EWDI2}_t - \text{EWDI2}_{t-12months}$
EWDI2_15mo	Change in EWDI2 over 15 months	$\text{EWDI2}_t - \text{EWDI2}_{t-15months}$
EWDI2_18mo	Change in EWDI2 over 18 months	$\text{EWDI2}_t - \text{EWDI2}_{t-18months}$
NDVI	Normalized difference vegetation index	$(\text{Band2} - \text{Band1})/(\text{Band2} + \text{Band1})$
NDVI_3mo	Change in NDVI over 3 months	$\text{NDVI}_t - \text{NDVI}_{t-3months}$
NDVI_6mo	Change in NDVI over 6 months	$\text{NDVI}_t - \text{NDVI}_{t-6months}$
NDVI_9mo	Change in NDVI over 9 months	$\text{NDVI}_t - \text{NDVI}_{t-9months}$
NDVI_12mo	Change in NDVI over 12 months	$\text{NDVI}_t - \text{NDVI}_{t-12months}$
NDVI_15mo	Change in NDVI over 15 months	$\text{NDVI}_t - \text{NDVI}_{t-15months}$
NDVI_18mo	Change in NDVI over 18 months	$\text{NDVI}_t - \text{NDVI}_{t-18months}$
MSI	Moisture stress index	Band 6/Band 2
MSI_3mo	Change in MSI over 3 months	$\text{MSI}_t - \text{MSI}_{t-3months}$
MSI_6mo	Change in MSI over 6 months	$\text{MSI}_t - \text{MSI}_{t-6months}$

MSI_9mo	Change in MSI over 9 months	$\text{MSI}_t - \text{MSI}_{t-9months}$
MSI_12mo	Change in MSI over 12 months	$\text{MSI}_t - \text{MSI}_{t-12months}$
MSI_15mo	Change in MSI over 15 months	$\text{MSI}_t - \text{MSI}_{t-15months}$
MSI_18mo	Change in MSI over 18 months	$\text{MSI}_t - \text{MSI}_{t-18months}$
ANOMtmn_1mo	Cumulative minimum temperature anomoly over preceding month	
ANOMtmn_3mo	Cumulative minimum temperature anomoly over preceding 3 months	
ANOMtmn_6mo	Cumulative minimum temperature anomoly over preceding 6 months	
ANOMtmn_9mo	Cumulative minimum temperature anomoly over preceding 9 months	
ANOMtmn_12mo	Cumulative minimum temperature anomoly over preceding 12 months	
ANOMtmn_15mo	Cumulative minimum temperature anomoly over preceding 15 months	
ANOMtmn_18mo	Cumulative minimum temperature anomoly over preceding 18 months	
ANOMtmx_1mo	Cumulative maximum temperature anomoly over preceding month	
ANOMtmx_3mo	Cumulative maximum temperature anomoly over preceding 3 months	
ANOMtmx_6mo	Cumulative maximum temperature anomoly over preceding 6 months	
ANOMtmx_9mo	Cumulative maximum temperature anomoly over preceding 9 months	
ANOMtmx_12mo	Cumulative maximum temperature anomoly over preceding 12 months	
ANOMtmx_15mo	Cumulative maximum temperature anomoly over preceding 15 months	
ANOMtmx_18mo	Cumulative maximum temperature anomoly over preceding 18 months	
AVGtmn_1mo	Average minimum temperature of preceding month	
AVGtmn_1mo_3mo	Change in AVGtmn over 3 months	$\text{AVG\_tmn}_t - \text{AVG\_tmn}_{t-3months}$
AVGtmn_1mo_6mo	Change in AVGtmn over 6 months	$\text{AVG\_tmn}_t - \text{AVG\_tmn}_{t-6months}$

AVGtmn_1mo_9mo	Change in AVGtmn over 9 months	$\text{AVG\_tmn}_t - \text{AVG\_tmn}_{t-9months}$
AVGtmn_1mo_12mo	Change in AVGtmn over 12 months	$\text{AVG\_tmn}_t - \text{AVG\_tmn}_{t-12months}$
AVGtmn_1mo_15mo	Change in AVGtmn over 15 months	$\text{AVG\_tmn}_t - \text{AVG\_tmn}_{t-15months}$
AVGtmn_1mo_18mo	Change in AVGtmn over 18 months	$\text{AVG\_tmn}_t - \text{AVG\_tmn}_{t-18months}$
AVGtmx_1mo	Average maximum temperature of preceding month	
AVGtmx_1mo_3mo	Change in AVGtmx over 3 months	$\text{AVG\_tmx}_t - \text{AVG\_tmx}_{t-3months}$
AVGtmx_1mo_6mo	Change in AVGtmx over 6 months	$\text{AVG\_tmx}_t - \text{AVG\_tmx}_{t-6months}$
AVGtmx_1mo_9mo	Change in AVGtmx over 9 months	$\text{AVG\_tmx}_t - \text{AVG\_tmx}_{t-9months}$
AVGtmx_1mo_12mo	Change in AVGtmx over 12 months	$\text{AVG\_tmx}_t - \text{AVG\_tmx}_{t-12months}$
AVGtmx_1mo_15mo	Change in AVGtmx over 15 months	$\text{AVG\_tmx}_t - \text{AVG\_tmx}_{t-15months}$
AVGtmx_1mo_18mo	Change in AVGtmx over 18 months	$\text{AVG\_tmx}_t - \text{AVG\_tmx}_{t-18months}$
PREC_1mo	Cumulative precipitation of the preceding month	
PREC_3mo	Cumulative precipitation of the preceding 3 months	
PREC_6mo	Cumulative precipitation of the preceding 6 months	
PREC_9mo	Cumulative precipitation of the preceding 9 months	
PREC_12mo	Cumulative precipitation of the preceding 12 months	
PREC_15mo	Cumulative precipitation of the preceding 15 months	
PREC_18mo	Cumulative precipitation of the preceding 18 months	
PREC_1mo_3mo	Change in PREC_1mo over 3 months	$\text{PREC\_1mo}_t - \text{PREC\_1mo}_{t-3months}$
PREC_1mo_6mo	Change in PREC_1mo over 6 months	$\text{PREC\_1mo}_t - \text{PREC\_1mo}_{t-6months}$
PREC_1mo_9mo	Change in PREC_1mo over 9 months	$\text{PREC\_1mo}_t - \text{PREC\_1mo}_{t-9months}$

PREC_1mo_12mo	Change in PREC_1mo over 12 months	$PREC\_1mo_t - PREC\_1mo_{t-12months}$
PREC_1mo_15mo	Change in PREC_1mo over 15 months	$PREC\_1mo_t - PREC\_1mo_{t-15months}$
PREC_1mo_18mo	Change in PREC_1mo over 18 months	$PREC\_1mo_t - PREC\_1mo_{t-18months}$

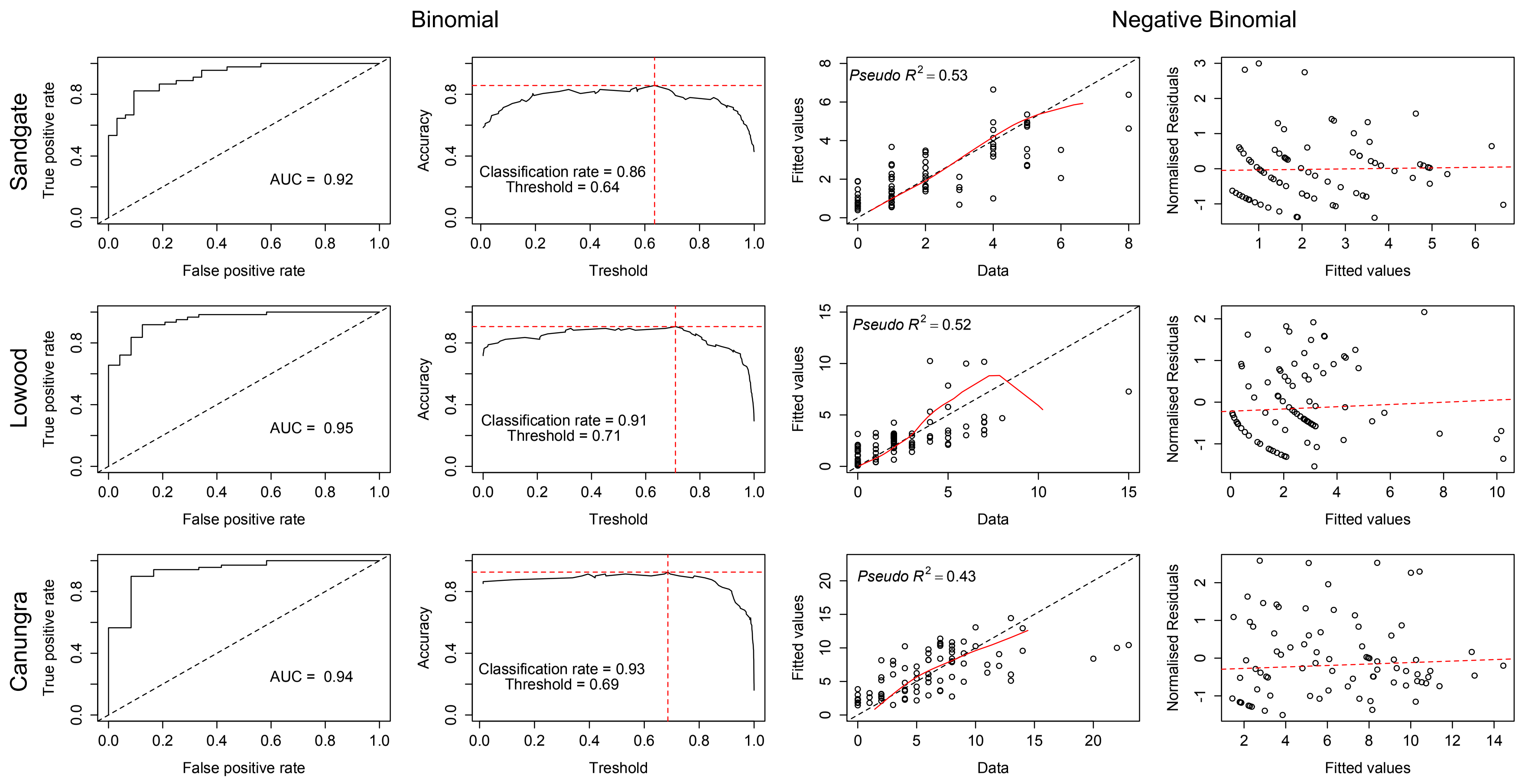

Figure A6: Model performance metrics of final models fitted to all data. The columns from left to right display: AUC, threshold of maximum classification accuracy, plots of fitted versus data values with Pseudo R2, and residuals versus fitted values.
